# Supplementary material for: Salivary glands regenerate after radiation injury through SOX2‐mediated secretory cell replacement
Source: EMBO Mol Med. 2018 Jan 15;10(3):e8051. doi: 10.15252/emmm.201708051 (PMC5840548; doi:10.15252/emmm.201708051)
Supplement: Supplementary file 1 — Appendix [file EMMM-10-e8051-s001.pdf]

Appendix Contents

Page	Content
1	Table S1: Sequences for mouse primers used for qPCR
2	Table S2: Sequences for human primers used for qPCR
3	Figure S1: Short-term ablation of SOX2+ cells reduces acinar cell replacement
4	Figure S2: Reinnervation 30 days following transection of the chorda tympani restores the acinar lineage

Table S1. Sequences for mouse primers used for qPCR

Gene target	Forward primer sequence	Reverse primer sequence
Aqp5	TCTACTTCTACTTGCTTTTCCCCTCCTC	CGATGGTCTTCTTCCGCTCCTCTC
Bak1	CAACCCCGAGATGGACAACTT	CGTAGCGCCGGTTAATATCAT
Bax	TGCTAGCAAACTGGTGCTCA	TCACGGAGGAAGTCCAGTGT
Bbc3	AGCAGCACTTAGAGTCGCC	CCTGGGTAAGGGGAGGAGT
Ccnd1	CATCCATGCGGAAAATCGTGG	AAGACCTCCTCTTCGCACTTC
Cdh1	GACTGGAGTGCCACCACCAAAGAC	CGCCTGTGTACCCTCACCATCGG
Cdkn1a	CCCCCAATCGCAAGGATTCTT	CTTGGTTCGGTGGGTCTGTC
Chrm1	TCCCAAGGCTCACCCAGATGTC	GCTCTGTGTGCTTTATTCTGTTGTTTCC
Chrm3	CATAGCACCATCCTCAACTCTACCAAG	GGGCATTTCTCTCTACATCCATAGTCC
Dta	ACCACGGGACTAAACCTGGTT	CTTCGTCAGTCCTGGATACGTC
Egfr	ACACTACGCCGCCTGCTTCAAGAG	ACTGTGCCAAATGCTCCCGAACCC
Gapdh	ACTCCACTCACGGCAAATTCAACGGCACAG	GGGTCTCGCTCCTGGAAGATGGTGATGGG
Kit	TGGTTGTGGTTGTTGTTGTTGTTG	GAAGGCTTGTTCCGAAGTGTAGAC
Krt5	TCCTGTTGAACGCCGCTGAC	CGGAAGGACACACTGGACTGG
Krt7	CGCCGCTGAGTGTGGACATCG	CTGGCTGCTCTTGGCTGACTTCTG
Krt8	GGAGGAGAGCAGGCTGGAGTC	TGGTGCGGCTGAAAGTGTTGG
Krt19	GCCACCTACCTTGCTCGGATTG	GTCTCTGCCAGCGTGCCTTC
MKi67	CATACCTGAGCCCATCACCA	GCTTTGCTGCATTCCGAGTA
Mist1	GCTGACCGCCACCATACTTAC	TGTGTAGAGTAGCGTTGCAGG
Muc19	CTGGGTCTGGAAGTAGAAGTA	TCTAAGCCACAGAAGGAGAT
Pmaip1	GCAGAGCTACCACCTGAGTTC	CTTTTGCGACTTCCCAGGCA
Rsp18	ATGGCCGTTCTTAGTTGGTG	GAACGCCACTTGTCCCTCTA
Sox2	CAGCATGTCCTACTCGCAGCAG	TGGAGTGGGAGGAAGAGGTAACC
Sox10	ATCAGCCACGAGGTAATGTCCAAC	ACTGCCCAGCCCGTAGCC
Trp53	CTCTCCCCCGCAAAAGAAAAA	CGGAACATCTCGAAGCGTTTA
Tubb3	CCAGAGCCATCTAGCTACTGACACTG	AGAGCCAAGTGGACTCACATGGAG
Vacht	GAGTGGGAGATGGGCATGGTTTGG	GCAGGCAGGTACGACGCAAGAG
Vip	TCCAGTGATAGGTACTCCATCTC	CATCCATAGCACACGCAGAA

Table S2. Sequences for human primers used for qPCR

Gene target	Forward primer sequence	Reverse primer sequence
AMY1	GGTGTTGCAGGGTTCAGAAT	TTTTAATTGGCTCACCACCC
AQP3	GTTTCTGTGTATGTGTATGTCTGCCTTT	CGTCCCACTGCTCCTACTTATGT
AQP5	CTGTCCATTGGCCTGTCTGTC	GGCTCATACGTGCCTTTGATC
CDH1	AGGTGACAGAGCCTCTGGATAGA	TGGATGACACAGCGTGAG AGA
CD44	CTGCCGCTTTGCAGGTGTA	CATTGTGGGCAAGGTGCTATT
CHRM1	ACCTCTATACCACGTACCTG	TGAGCAGCAGATTCATGACG
CHRM3	ATCGGTCTGGCTTGGGTC	CCCGGAGGCACAGTTCTC
EGFR	TGGCAGGTACAGTAGGATAA	CAAGTCAGTCTAACGCTCAT
GAPDH	CAGCCTCAAGATCATCAGCA	TGTGGTCATGAGTCCTTCCA
GFRA	CTTCACAGAGCTCACGACAAA	TCAGCATCAGGACAGACAGC
KIT	GCAGAGGAAGTGGAAGGCATCAG	TCAGTGAGACAGTAGCATTATGGAAGGT
KRT5	CGTGCCGCAGTTCTATATTCT	ACTTTGGGTTCTCGTGTCAG
KRT7	TCCGCGAGGTCACCATTAAC	GCTCTGTCAACTCCGTCTCAT
KRT8	AAGGATGCCAACGCCAAGTT	CCGCTGGTGGTCTTCGTATG
KRT19	GTCTGCCTCCAAGGTCCTCTGA	TCTACCCAGAAGACACCCTCCAAA
MIST1	CGGATGCACAAGCTAAATAACG	GCCGTCAGCGATTTGATGTAG
NGF	CAACAGGACTCACAGGAGCA	GTCTGTGGCGGTGGTCTTAC
NRTN	CCCTGCCTGTGATGCCATTCTC	GAGCCGATGACAAGGTCCAGACT
SOX2	TGGCGAACCATCTCTGTGGT	GGAAAGTTGGGATCGAACAAAAGC
SOX10	TCATCCCTTCAATGCCCCCT	TGCGTCTCAAGGTCATGGAGG
TH	GTGCTAAACCTGCTCTTCTC	CGTCTCAAACACCTTCACAG
TUBB3	CGAAGCCAGCAGTGTCTAAA	GGAGGACGAGGCCATAAATA
VIP	CTTGAGTCTCTTATGGGAAAACGTGT	GTGAAGACTGCATCTGAGTGACG

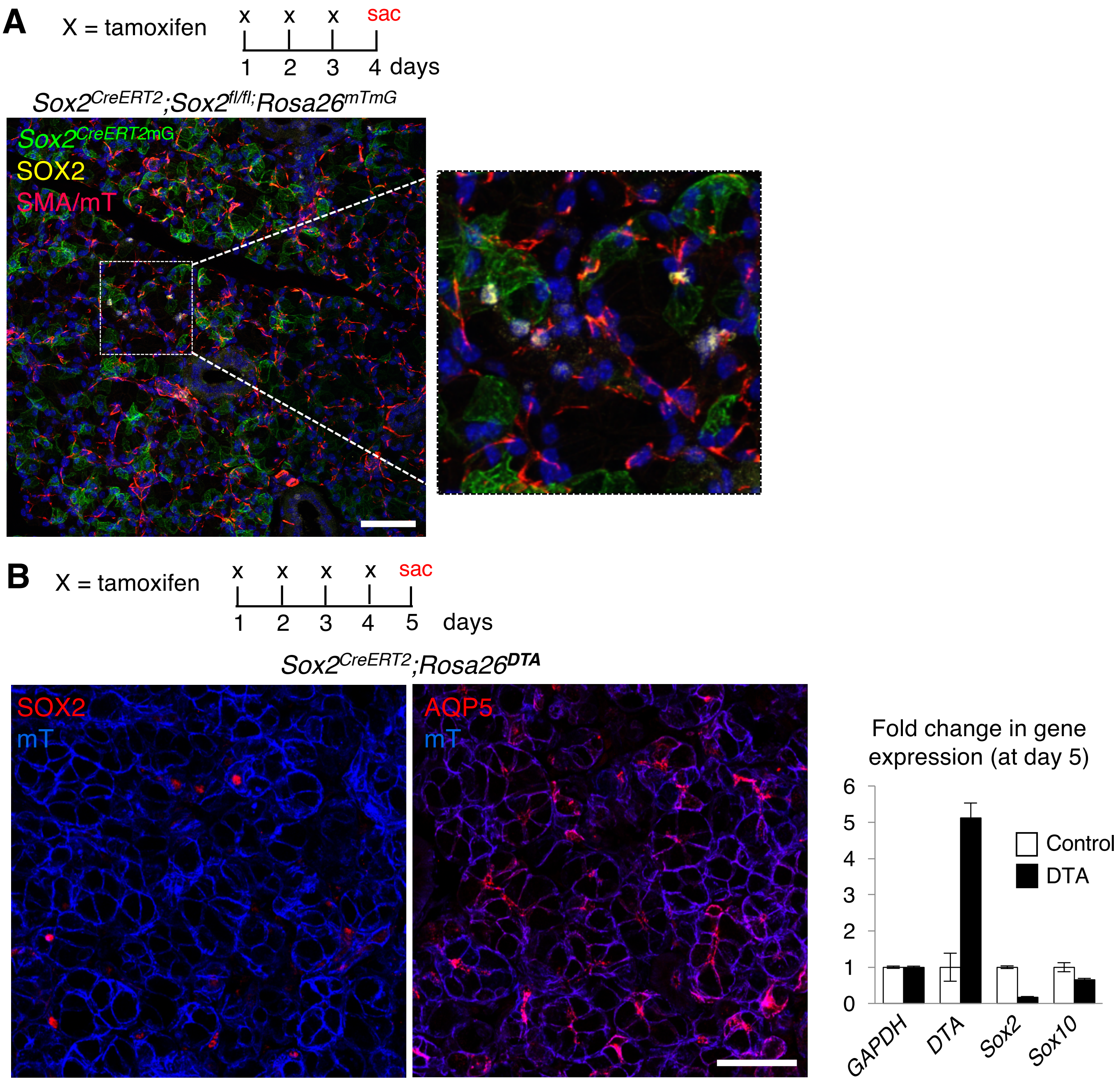

Figure S1. Short-term ablation of SOX2+ cells reduces acinar cell replacement. (A) SOX2+ cells were ablated in SLG of *Sox2*^{CreERT2};*Sox2*$^{fl/fl}$; *Rosa26*$^{mTmG/+}$ mice over 3 days and SLG analyzed on day 4 (see schematic). Sections of *Sox2*^{CreERT2};*Sox2*$^{fl/fl}$; *Rosa26*$^{mTmG/+}$ SLG were immunostained for SOX2 and myoepithelial cells (α-smooth muscle actin; SMA). Scale bar = 50 μm (**B**) SOX2+ cells were ablated in SLG of *Sox2*^{CreERT2}*Rosa26*^{DTA}; *Rosa26*$^{mTmG/+}$ mice over 4 days and SLG analyzed on day 5 (see schematic). Sections of *Sox2*^{CreERT2}*Rosa26*^{DTA} SLG were immunostained for SOX2 and acinar cells (aquaporin 5; AQP5) or subjected to qPCR. Scale bar = 50 μm.

Data information: Data in **B** were normalized to *Gapdh* and the wild-type control. Data is mean+S.D. (n=1, 3 technical replicates).

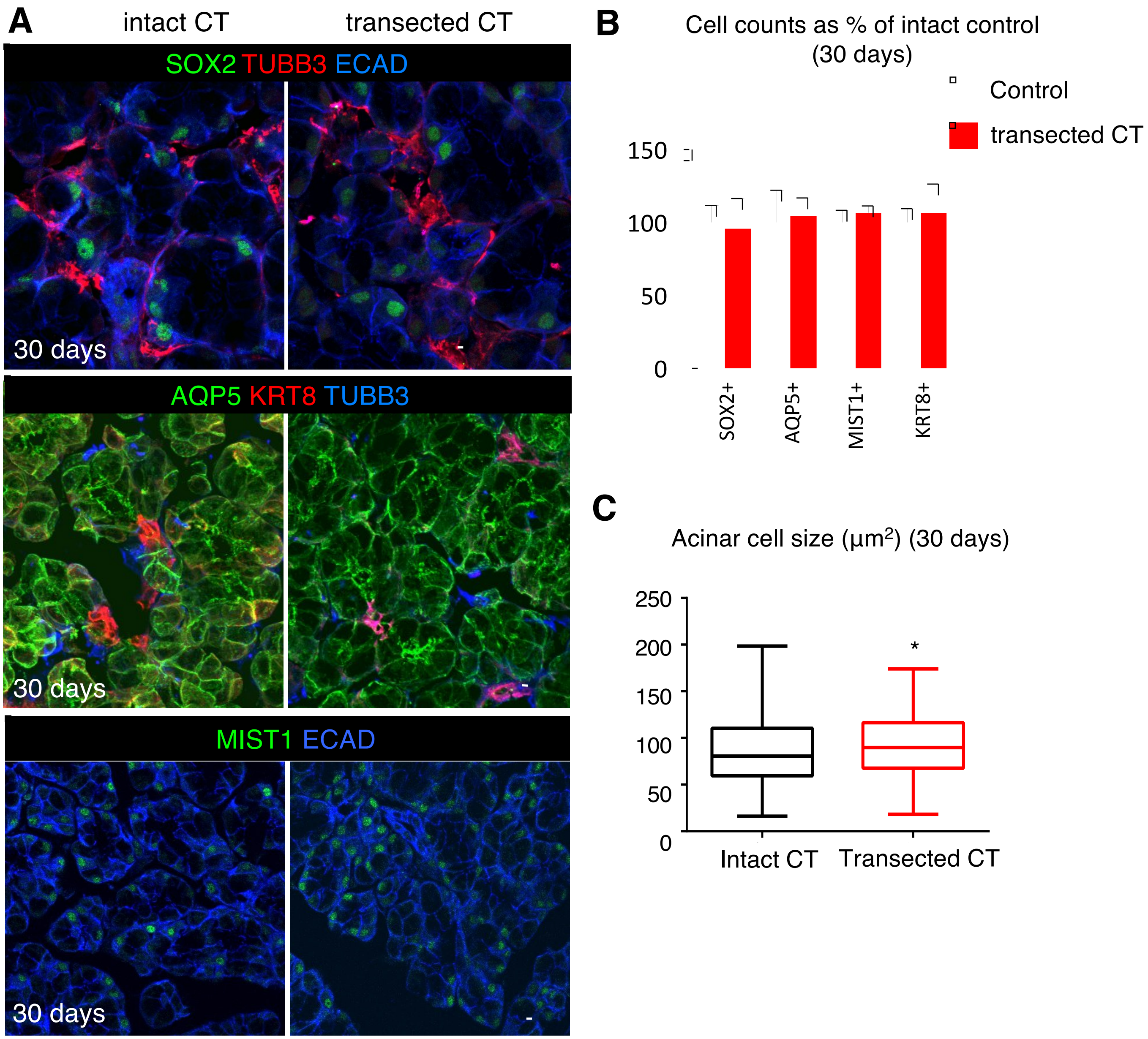

Figure S2. Reinnervation 30 days following transection of the chorda tympani restores the acinar lineage (A-C) SLG were immunostained for SOX2, TUBB3, AQP5, MIST1 and ECAD **(A)** and numbers of SOX2+, AQP5+, MIST1+ and KRT8+ cells **(B)** as well as acinar cells size **(C)** was measured 30 days after denervation. n = 5 mice per time point per condition. Cells were counted in 3-4 fields of view per animal. Data information: Data in **B** and **C** n=5. Data in **B** are mean+S.E.M and were analyzed using a one-way analysis of variance test, data in **C** are a box and whisker plot of n=5 mice, showing mean+S.E.M. and were analyzed using students *t*-test. *p = 0.0498.
