# Supplementary material for: Salivary glands regenerate after radiation injury through SOX2‐mediated secretory cell replacement
Source: EMBO Mol Med. 2018 Jan 15;10(3):e8051. doi: 10.15252/emmm.201708051 (PMC5840548; doi:10.15252/emmm.201708051)
Supplement: Supplementary file 2 — Expanded View Figures PDF [file EMMM-10-e8051-s002.pdf]

Expanded View Figures

Figure EV1. SOX2 marks a subset of acinar cells that replenish acini.

A Wild-type murine PG stained for SOX2, ECAD, and nuclei. Scale bar is 50 μm.
B The percentage of acinar $SOX2^+$ and $SOX2^-$ cells that are $Ki67^+$ were counted using FACS and shown as a percentage of total $AQP5^+SOX2^+$ or $AQP5^+SOX2^-$ cells.
C The % of $SOX2^+$ acinar cells that are either $CyclinD1^+$ or $CyclinD1^-$.
D Schematic of *Rosa26*^{mTmG} Cre-mediated gene excision (adapted from Muzumdar *et al*, 2007).
E Representative image of *Sox2* lineage-traced SLG. Cre-mediated recombination was induced in *Sox2*^{CreERT2};*Rosa26*^{mTmG} mice and SLG analyzed 14 or 30 days later by immunostaining for SOX2. Scale bar = 25 μm.
F Representative images of *Kit* lineage-traced SLG and SMG. Cre-mediated recombination was induced in *Kit*^{CreERT2};*Rosa26*^{mTmG} mice and SMG/SLG analyzed 14 days and 6 months later. Tissue was stained with AQP5 to mark acinar cells and KRT8 to mark intercalated duct cells. Scale bar = 25 μm. mT = membrane-bound Tomato.

Data information: Data in (B), SLG were pooled from n = 2 mice (85,000 events). Data in (C) were calculated from three non-consecutive fluorescent sections of each SLG from n = 3 mice with individual values plotted. Error bars show mean ± SD.

© 2018 The Authors

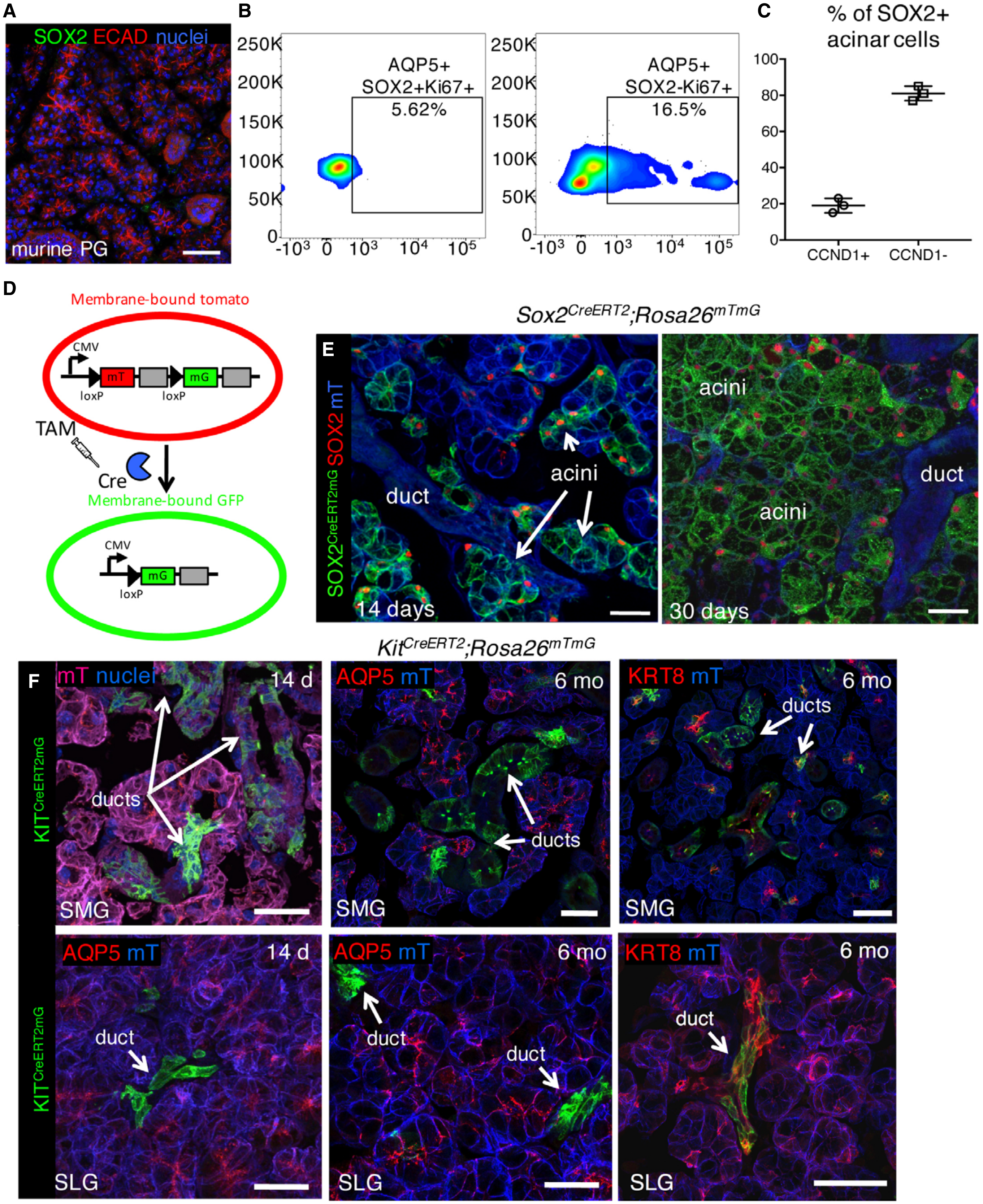

Figure EV1.

 © 2018 The Authors

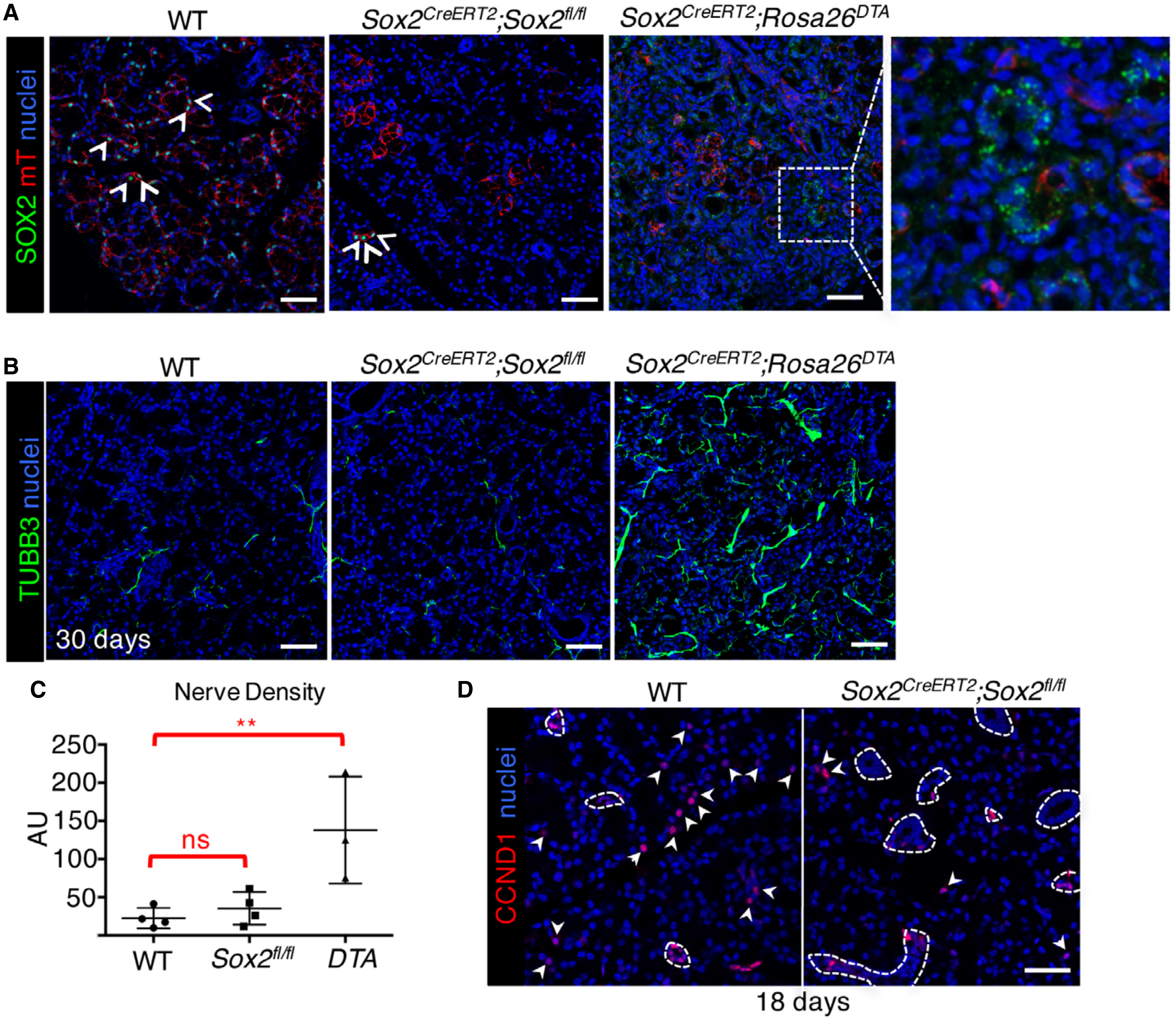

Figure EV2. Ablation of *Sox2* or SOX2^{+} cells reduces acinar cell replacement despite the presence of nerves.

A–C *Sox2* or SOX2^{+} cells were ablated in SLG of *Sox2^{CreERT2}; Sox2$^{fl/fl}$; Rosa26$^{mTmG/+}$* mice (Fig 2A; see schematic) or *Sox2^{CreERT2}; Rosa26^{DTA}; Rosa26$^{mTmG/+}$* mice (Fig 2B; see schematic). (A, B) Sections of WT, *Sox2^{CreERT2};Sox2$^{fl/fl}$*, and *Sox2^{CreERT2};Rosa26^{DTA}* SLG were immunostained for SOX2 or TUBB3 and nuclei. White arrowheads indicate SOX2^{+} cells. White dotted square is magnified in the image to the right to highlight that there are few SOX2^{+} cells remaining in tissue and that non-nuclear (green) staining is suggestive of debris. Scale bar = 50 μm. (C) Raw integrated density of nerves was calculated using ImageJ.

D WT or *Sox2^{CreERT2};Sox2$^{fl/fl}$* SLG immunostained for cyclin D1 (CCND1) and nuclei. Dashed lines = ducts; arrowheads = CCND1^{+} acinar cells. Scale bar = 50 μm.

Data information: Data in (C), WT n = 4, *Sox2$^{fl/fl}$* n = 4, *DTA* n = 3. Individual values were plotted, as means + SD, and data were analyzed using a one-way analysis of variance with *post hoc* Dunnett's test. **P = 0.0091. Data in (D) are a representative image from n = 4 mice.

Figure EV3. Transection of the chorda tympani depletes acinar cells at 7 days.

A–G (A, C, D, G) Control and nerve transected SLG were immunostained 7 days after denervation for tyrosine hydroxylase (TH; A), SOX2 and TUBB3 (C), KRT5 (D), caspase-3 (CASP3; G), epithelial cells (ECAD), and nuclei. Quantification of the size of acinar cells (B) in adult wild-type (WT) SLG with intact or transected chorda tympani (CT) 7 days after denervation. Scale bars in (A, C, D, G) = 25 μm. (E, F) Recombination was induced in $Sox2^{CreERT2}$;$Rosa26^{mTmG}$ mice 24 h before nerve transection and SLG traced for 15 days before being immunostained for TUBB3. The percentage of GFP^{+} and mT^{+} acinar cells in control and transected glands are shown in (F). Scale bar in (E) = 25 μm. (H) Fold change in expression of genes involved in cell cycle and apoptosis 7 days after denervation, compared to intact control. Dashed line denotes the intact control.

Data information: Data in (B, F, and H) $n = 5$. Data in (B) are a box and whisker plot of $n = 5$ mice, showing means (horizontal line), upper and lower quartiles (box) and upper and lower values (whiskers) and were analyzed using Student's *t*-test. ***$P = 0.00000347$. Data in (F) are means + SD and were analyzed by Student's *t*-test. %GFP^{+} ***$P = 0.0000208$, %mT^{+} ***$P = 0.0000208$. Data in (H) were normalized to *Rsp18* and the intact control (dashed line). *Ccnd1* *$P = 0.0477$.

© 2018 The Authors

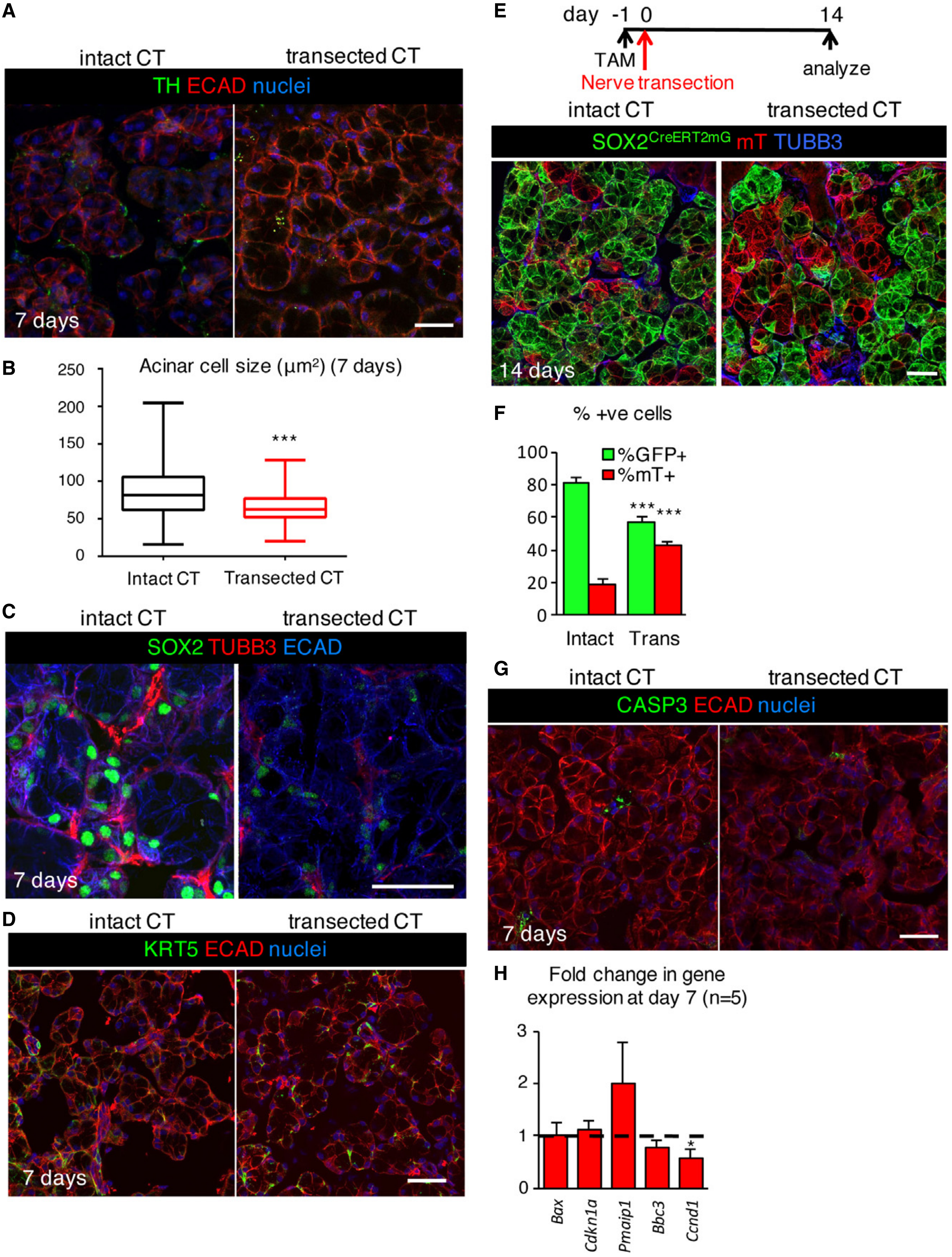
A
intact CT
transected CT
TH ECAD nuclei
7 days
B
Acinar cell size (μm²) (7 days)
250
200
150
100
50
0
***
Intact CT
Transected CT
C
intact CT
transected CT
SOX2 TUBB3 ECAD
7 days
D
intact CT
transected CT
KRT5 ECAD nuclei
7 days
E
day -1 0 14
TAM
Nerve transection
analyze
intact CT
transected CT
SOX2CreERT2mG mT TUBB3
14 days
F
% +ve cells
100
80
60
40
20
0
%GFP+
%mT+
*** ***
Intact Trans
G
intact CT
transected CT
CASP3 ECAD nuclei
7 days
H
Fold change in gene expression at day 7 (n=5)
3
2
1
0
*
Bax
Cdkn1a
Pmaip1
Bbc3
Ccnd1

Figure EV3.

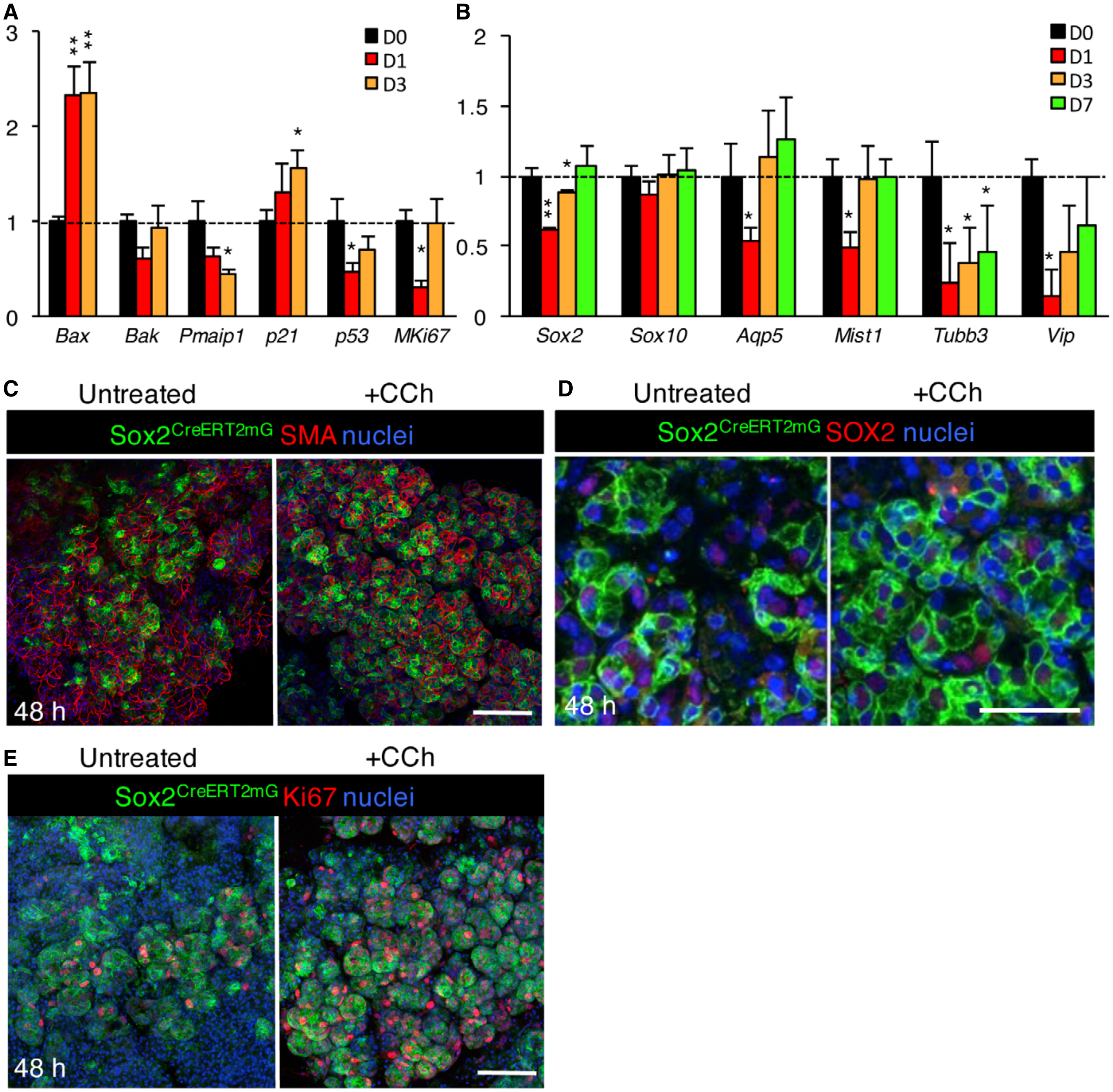

Figure EV4. IR induces cellular damage and a loss of nerves and SOX2 and SOX2$^+$ progenitors can replenish mouse acinar cells in response to cholinergic mimetics.

A, B Murine SLG was analyzed for transcriptional changes by qPCR at days 0, 1, 3, and 7 days following 10 Gy of IR.

C–E *Sox2*^{CreERT2};*Rosa26*^{mTmG} murine SLG explants cultured *ex vivo* for 48 h and immunostained for smooth muscle actin (SMA, C), SOX2 (D) or Ki67 (E) and nuclei. Recombination was induced 24 h before SLG was harvested for culture. Scale bar = 50 µm.

Data information: Data in (A and B) were normalized to *Rsp29* and the day 0 control. Data in (A and B) (n = 3 per time point) are means + SEM and were analyzed using a one-way analysis of variance with a *post hoc* Dunnett's test. *Bax* (D1) $^{**}P$ = 0.00826, *Bax* (D3) $^{**}P$ = 0.0871, *Pmaip1* (D3) $^{*}P$ = 0.0369, *p21* (D3) $^{*}P$ = 0.0481, *p53* (D1) $^{*}P$ = 0.0337, *MKi67* (D1) $^{*}P$ = 0.0319, *Sox2* (D1) $^{**}P$ = 0.0072, *Sox2* (D3) $^{*}P$ = 0.0418, *Aqp5* (D1) $^{*}P$ = 0.0421, *Mist1* (D1) $^{*}P$ = 0.0432, *Tubb3* (D1) $^{*}P$ = 0.0467, *Tubb3* (D3) $^{*}P$ = 0.0470, *Tubb3* (D7) $^{*}P$ = 0.0489, *Vip* (D1) $^{*}P$ = 0.0402. Images in (C, D, and E) are representative of three experiments, n = 3 SLG fragments per experiment.

 © 2018 The Authors

Figure EV5. Muscarinic activation is sufficient to increase *SOX2* expression and the acinar lineage in human adult salivary gland.

A Human salivary gland obtained from healthy individuals (no IR; submandibular) or patients who received radiation therapy for head and neck cancer (IR) were subjected to qPCR.
B Representative images of adult human (h) salivary gland (non-IR, 22–31 years; SMG) immunostained for endogenous SOX10, MIST1, EGFR, CD44, KRT7, AQP3, KRT5, ECAD, and nuclei. Single arrowheads indicate SOX10-expressing acinar and ductal cells. Scale bars = 50 μm.
C Quantification of number of $Ki67^+$ cells in nerve co-culture (representative images shown in Fig 7C).
D Adult human salivary gland explants (SMG or PG) from four individual patients (healthy, non-IR) were cultured for 4 h ± 200 nM CCh. Pooled data presented in Fig 7E.

Data information: Data in (A) n = 11 for no IR and n = 7 for IR; 30–85 years. Data were normalized to *GAPDH* with individual values plotted and analyzed using a Student's t-test with a false discovery rate set to 0.05. Error bars show mean ± SD. *TUBB3* P = 0.346, *VIP* P = 0.461, *GFRA* P = 0.002, *TH* P = 0.433. Data in (C) are n = 3 and mean + SEM and were analyzed using a Student's t-test. *P = 0.0151. Data in (D) (n = 4 separate individuals) were normalized to *GAPDH* expression and salivary gland from the same individuals cultured with no CCh (control; black dashed line) and run in triplicate and are presented as mean + SD.

© 2018 The Authors

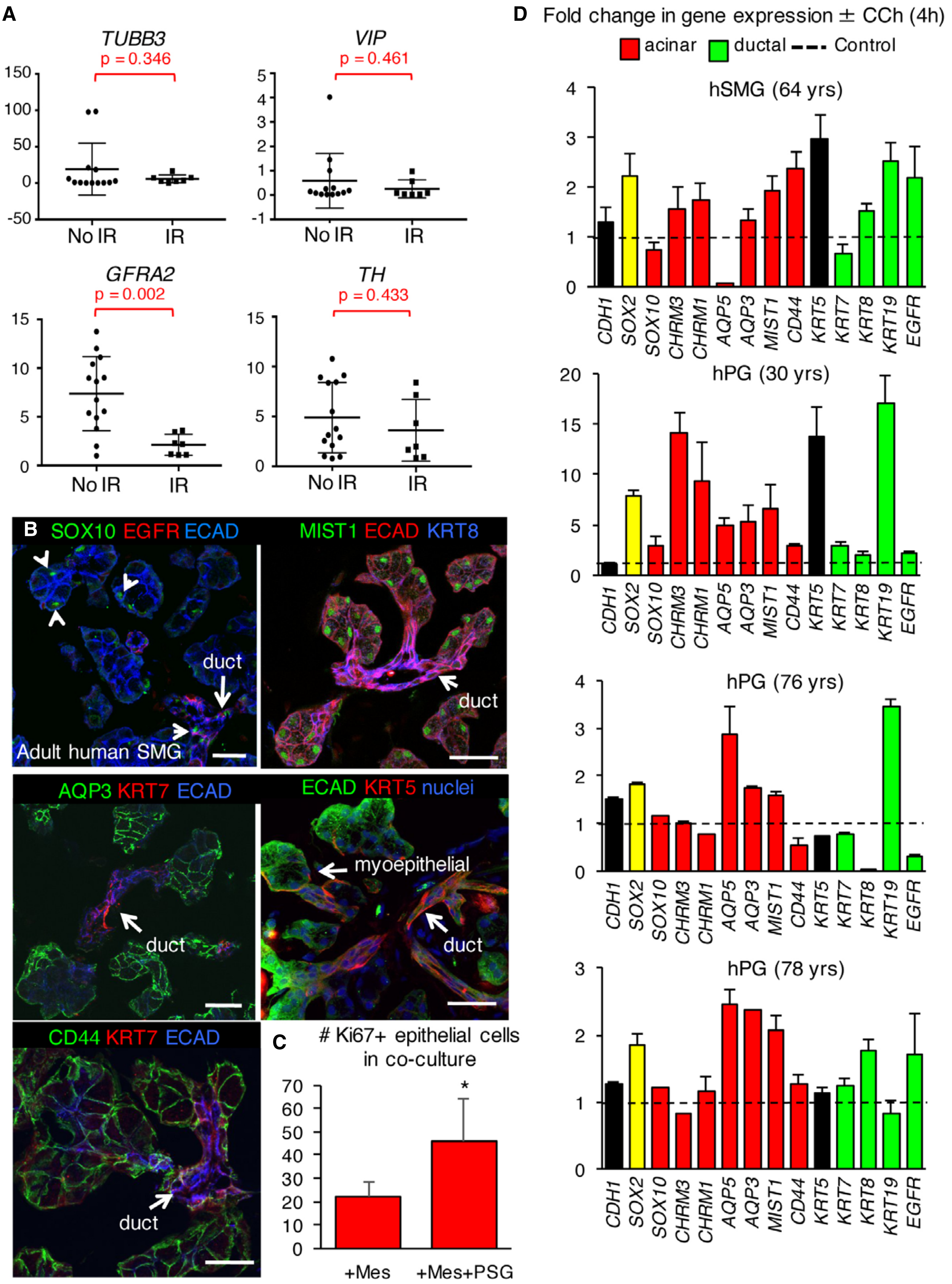

Figure EV5.

 © 2018 The Authors
